# Engineered biosorbents of pomelo (*Citrus maxima* (Burm.f.) Merr) peels modified with zinc oxide and titanium dioxide for methylene blue dye sorption

**DOI:** 10.1038/s41598-024-56499-z

**Published:** 2024-03-08

**Authors:** Pornsawai Praipipat, Pimploy Ngamsurach, Pratchayaporn Srirat, Punjaporn Chaiphumee

**Affiliations:** 1 Department of Environmental Science, Faculty of Science, Khon Kaen University, Khon Kaen, 40002 Thailand; 2 Environmental Applications of Recycled and Natural Materials (EARN) Laboratory, Khon Kaen University, Khon Kaen, 40002 Thailand

**Keywords:** Citrus peel, Metal oxide, Bead, Adsorption, Dye, Engineering, Materials science

## Abstract

The pomelo-doped zinc oxide beads (PZB), pomelo-doped titanium dioxide beads (PTB), and pomelo-doped zinc oxide and titanium dioxide beads (PZTB) were synthesized for sorbing methylene blue (MB) dye. Their characterizations were explored by X-Ray Diffractometer (XRD), Field Emission Scanning Electron Microscopy and Focus Ion Beam (FESEM-FIB), Energy Dispersive X-Ray Spectrometer (EDX), and Fourier Transform Infrared Spectroscopy (FT-IR). In addition, their sorbent efficiencies for sorbing MB dye were investigated through batch experiments, sorbent reusability studies, sorption isotherms, kinetics, and thermodynamic studies. They were crystalline phases presenting the specific peaks of zinc oxide (ZnO) or titanium dioxide (TiO_2_). Their surfaces had lamella structures with coarse surfaces, and they also found specific structures of ZnO or TiO_2_ on the surfaces. Zn–O or Ti–O–Ti was also detected in PZB or PTB or, PZTB depending upon metal oxide types added into pomelo beaded sorbents. For batch experiments, they could adsorb MB dye of more than 86%, and PZTB showed the highest MB dye removal efficiency. In addition, they could be reused for more than three cycles with high MB dye sorptions of more than 72%. They corresponded to Freundlich and pseudo-second-order kinetic models. Moreover, the increasing temperature affected their decreasing MB dye sorptions which were exothermic processes.

## Introduction

Methylene blue (MB) dye is one of the dyes that is popularly used in many industries of textile, printing, plastic, printing paper, rubber, cosmetics, medicine, and food, so wastewater from these industries may contaminate MB dye molecules causing toxicity to water quality, ecological system, and living organisms if they are not treated before releasing to the environment^1^. MB dye is a cationic azo dye that is persistent because of non-biodegradable, toxicity, and bioaccumulation. The releasing of MB dye into the water body causes the decrease of oxygen and obstacle photosynthesis of aquatic organisms, and it also threatens human health from human consumption by causing dysfunctional systems including human cancers^2,3^. Therefore, it is recommended to treat the wastewater-contaminated MB dye to protect human consumption and the environment.

The sorption method has been popularly used to eliminate many environmental pollutants of heavy metals and dyes in wastewater because it is an efficient method with offers easy operation, suitable cost, and many alternative sorbents. The choices of sorbent may be from commercial, agricultural, industrial, and waste depending upon the availability of raw material, sorption capacity, and target pollutant that the research is looking for. Fruit waste sorbents have been widely used as biosorbents for removing many cationic dyes in wastewater shown in Table 1 because they consist of chemical elements and functional groups that can catch up with dye ions. In addition, using them as dye sorbents helps to reduce the huge amount of fruit waste for promoting waste good management. In Table 1, the brilliant green and rhodamine 6G dyes are sorbed by lemon, rock melon, and litchi^4–6^, and the basic red 46 and MB dyes are removed by cactus fruit, pomegranate, *Dialium guineense*, atemoya, and pomelo^7–11^. Among those sorbents, the pomelo peels illustrated the highest sorption capacity (*q*_m_) than other sorbents, so it is interesting to use pomelo peels as dye sorbents. However, the sorbent improvement of pomelo peels also needs to be investigated for dealing with high dye concentrations in wastewater including the specific target pollutant.

**Table 1:** Fruit sorbents for removing cationic dyes.

Waste peels	Dyes	Dose (g)	Time (min)	Temp. (°C)	pH	Conc. (mg/L)	Volume (mL)	*q*_m_ (mg/g)	References
Non-modification									
Lemon	Brilliant green	0.10	90	24	5.4	50–200	100	150.00	^4^
Rock melon	Brilliant green	0.02	120	25	–	0–1000	10	118.00	^5^
*Litchi chinensis*	Rhodamine 6G	0.30	–	32	8	20–50	50	6.67	^6^
Cactus fruit	Basic red 46	1.00	–	25	6	20–200	500	136.80	^7^
Pomegranate	Basic red 46	1.00	60	25	7	20–200	500	120.48	^8^
*Dialium guineense*	Methylene blue	0.01	480	25	7	50–900	50	50.62	^9^
Atemoya	Methylene blue	0.02	720	45	7	10–200	10	190.18	^10^
Pomelo	Methylene blue	0.20	120	30	10	5–1000	50	218.50	^11^
Modification									
*Citrus macroptera* biochar (350 °C)	Methylene blue	0.004	75	25	8	20–80	20	139.70	^12^
Pomelo peel biochar (500 °C)	Methylene blue	0.100	1440	25	7	20–200	100	28.65	^13^
Pomelo peel biochar (600 °C)	Methylene blue	0.002	–	25	7	0.5–10	100	154.71	^14^
Pomelo peel biochar (600 °C) modified by nitric acid	Methylene blue	0.002	–	25	7	0.5–10	100	176.20	^14^
Pomelo peel biochar (600 °C) modified by sulfuric acid	Methylene blue	0.002	–	25	7	0.5–10	100	209.16	^14^
Pomelo peel modified by iron(III) chloride	Methylene blue	1.000	60	–	12	20–300	250	79.47	^15^
Papaya peel activated by phosphoric acid	Methylene blue	0.300	90	30	6	10–50	100	46.95	^16^
Watermelon modified by sulphuric acid	Methylene blue	0.080	180	30	5.6	50–400	100	200.00	^17^
Pomelo peel modified by polydopamine	Methylene blue	0.005	360	–	–	–	20	434.78	^18^

Many previous studies have modified fruit sorbents by treating thermal and chemical to increase sorbent capacity for removing MB dye shown in Table 1. The *citrus macroptera* and pomelo peels were modified by the thermal treatment at 350–600 °C^12–14^, whereas pomelo peels were also modified by thermal and chemical treatments^14,15^. For chemical treatment, phosphoric and sulfuric acids are used to modify papaya and watermelon peels^16,17^. Furthermore, the pomelo peels are modified by polydopamine^18^. Although pomelo peels with thermal or chemical treatment are used for removing MB dye in Table 1, no one does the beaded pomelo peels with metal oxide modification to eliminate it. Therefore, this study was the first attempt to synthesize pomelo peels with zinc oxide and titanium dioxide for MB dye sorption.

In this study, three pomelo beaded sorbents which were pomelo-doped zinc oxide beads (PZB), pomelo-doped titanium dioxide beads (PTB), and pomelo-doped zinc oxide and titanium dioxide beads (PZTB) were synthesized for MB dye sorptions. Several characterized techniques of X-Ray Diffractometer (XRD), Field Emission Scanning Electron Microscopy and Focus Ion Beam (FESEM-FIB), Energy Dispersive X-Ray Spectrometer (EDX), and Fourier Transform Infrared Spectroscopy (FT-IR) were used for determining their crystalline structures, surface morphologies, chemical elements, and functional groups, and their points of zero charge were also studied. Their MB dye removal efficiencies were examined by batch tests and sorbent reusability studies. The sorption isotherms, kinetics, and thermodynamic studies were also explored.

## Material and method

### Raw material preparation

Pomelo (*Citrus maxima* (Burm.f.) Merr) peels were wastes obtained from a fruit market in Khon Kaen province, Thailand. The contaminations in pomelo peels were eliminated by tap water washing, and they were cut into small pieces and dried in a hot air oven (Binder, FED 53, Germany) at 100 °C for 24 h. They were blended and sieved in size of 125 µm. Then, they were kept in a desiccator before use called pomelo powder (PP).

### Chemicals

Zinc oxide (ZnO) (AR, QRëc, New Zealand), titanium dioxide (TiO_2_) (AR, Loba, India), sodium alginate (NaC_6_H_7_O_6_) (AR, Merck, Germany), calcium chloride dihydrate (CaCl_2_.2H_2_O) (AR, RCI Labscan, Thailand), sodium chloride (NaCl) (AR, RCI Labscan, Thailand), methylene blue (MB) dye (C_16_H_18_ClN_3_S.xH_2_O (x = 2–3)) (AR, QRëc, New Zealand), 0.1 M or 0.5 M of 37% hydrochloric acid (HCl) (AR, RCI Labscan, Thailand) and 0.1 M NaOH (RCI Labscan, Thailand) were used in this study without purification. The water used in this study was the deionized water (DI water) produced from ELGA, Ultra GE MK2 purification system with a resistivity of 18.2 MegaOhmn-cm and conductivity of 0.055 Microsiemens/cm.

### Sorbent synthesis

**Figure 1:**
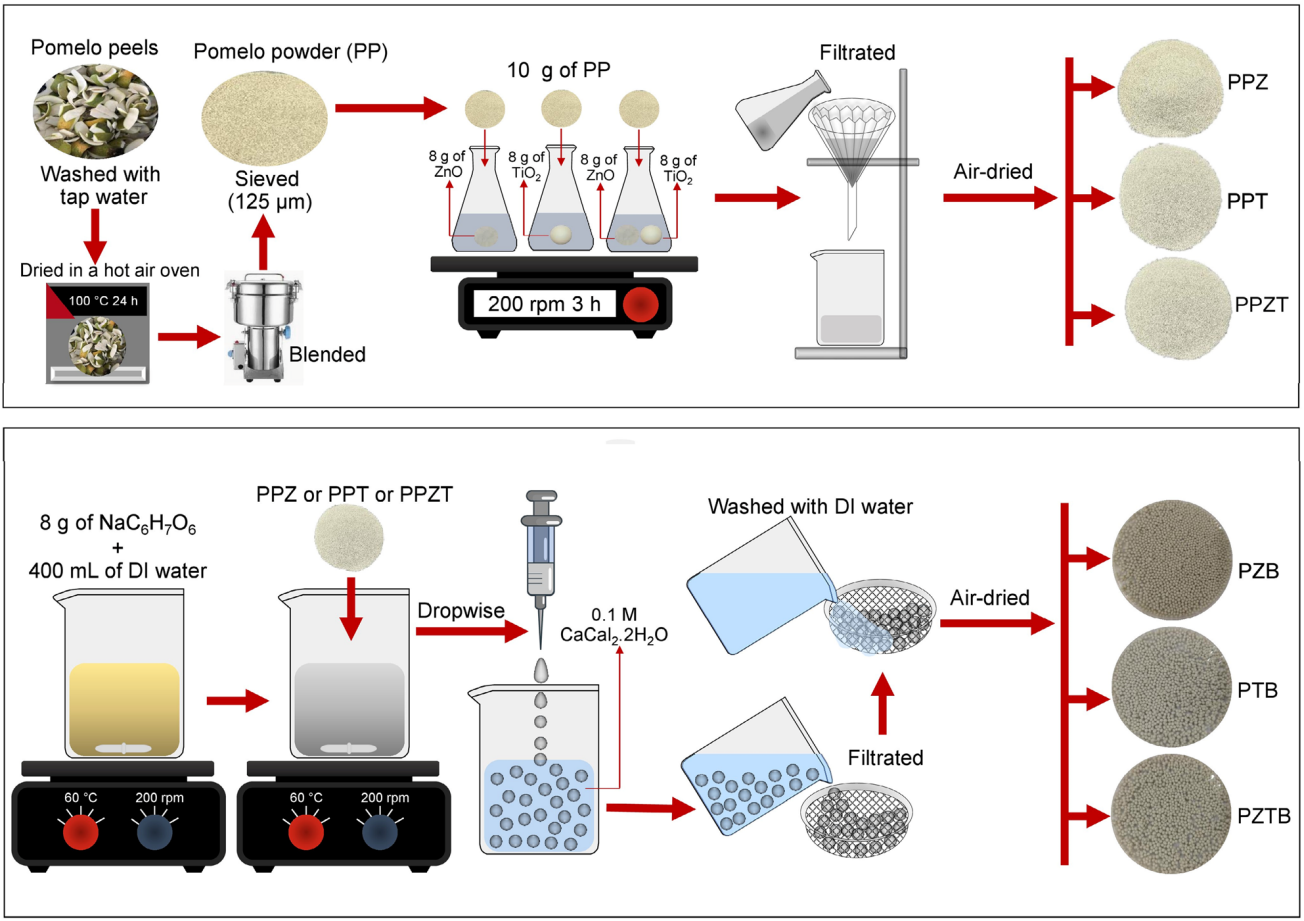
The synthesis method of PZB, PTB, and PZTB in a schematic flow diagram.

The sorbent synthesis of pomelo-doped zinc oxide beads (PZB), pomelo-doped titanium dioxide beads (PTB), and pomelo-doped zinc oxide and titanium dioxide beads (PZTB) was demonstrated in the schematic flow diagram in Fig. 1. Firstly, 10 g of PP was added to the ZnO or TiO_2_, or ZnO + TiO_2_ solution which was prepared from 8 g of ZnO or 8 g of TiO_2_, or 4 g of ZnO + 4 g of TiO_2_ in 160 mL of deionized (DI) water. Then, it was mixed by an orbital shaker (GEL, 3020, Germany) of 200 rpm for 3 h, filtrated, and air-dried called pomelo powder doped ZnO (PPZ) or pomelo powder doped TiO_2_ (PPT), or pomelo powder doped ZnO and TiO_2_ (PPZT). After that, it was added to the sodium alginate solution which was prepared from 8 g of NaC_6_H_7_O_6_ dissolved in 400 mL of DI water. It was homogeneously mixed by a hot plate (Ingenieurbüro CAT, M. Zipperer GmbH, M 6, Germany) with a magnetic stirrer of 200 rpm at 60 °C. Then, it was contained in a 10 mL syringe with a needle of 1.2 × 25 mm and was added dropwise into 0.1 M CaCl_2_.2H_2_O solution. After that, they were filtrated, washed with DI water, air-dried for 24 h, and kept in a desiccator before use called pomelo-doped zinc oxide beads (PZB) or pomelo-doped titanium dioxide beads (PTB), or pomelo-doped zinc oxide and titanium dioxide beads (PZTB).

### Sorbent characterizations

The characterizations of PZB, PTB, and PZTB on crystalline structures by X-Ray Diffractometer (XRD) analysis at 2ϴ of 5–80°, sorbent structures and chemical elements by Field Emission Scanning Electron Microscopy and Focus Ion Beam (FESEM-FIB) with Energy Dispersive X-Ray Spectrometer (EDX) by using 10 kV measurement which the samples were coated with gold, and chemical functional groups by Fourier Transform Infrared Spectroscopy (FT-IR) from 4000 to 600 cm^−1^ with a resolution of 4 cm^−1^ and 32 scans are investigated.

### The point of zero charge

The point of zero charge method is referred from the study of Praipipat et al.^19^ to determine what pH value represents its zero charge which might occur in MB dye sorption by each sorbent. In summary, 0.1 g of PZB or PTB, or PZTB was added to 50 mL of 0.1 M NaCl solution at pH 1–12 and shaken by an orbital shaker at 150 rpm for 24 h. Then, the final pH was measured by a pH meter (Mettler Toledo, Seven2Go pH with InLab Expert Go-ISM, Switzerland), and the intersection point between ∆pH (pH_final_ – pH_initial_) and pH presented pH_pzc_.

### Batch experiments

MB dye removal efficiencies of PZB, PTB, and PZTB were investigated by batch tests on dose (0.02–0.5 g), contact time (1–12 h), temperature (20–50 °C), pH (5–12), concentration (25–200 mg/L) with the control conditions of MB dye concentration of 100 mg/L, shaking speed of 150 rpm, and sample volume of 100 mL to determine their optimum conditions obtained the highest MB dye removal efficiency. The triplicate samples were applied to verify the results. MB dye concentration was measured by a UV–vis spectrophotometer (Specord 200 Plus Double-beam, Analytikjena, Germany), and the MB dye removal efficiency in percentage and the sorption capacity were calculated following Eqs. (1) and (2).

1 $${\text{MB dye removal efficiency}}\left( \% \right) = \, (C_{0} - C_{{\text{e}}} )/C_{0} \times { 1}00$$

where *C*_0_ is the initial MB dye concentration (mg/L), and *C*_e_ is the final MB dye concentration (mg/L).

2 $${\text{The}}\,{\text{sorption}}\,{\text{capacity}}\,\left( {q_{{\text{e}}} } \right) = \,\left( {\left( {C_{0} - C_{{\text{e}}} } \right) \times V} \right)/M$$

where *V* is the sample volume (L), and *M* is the mass of sorbent (g).

### Sorbent reusability studies

The method of sorbent reusability studies is referred from the study of Praipipat et al.^20^ to investigate the sorbent stability with reuse through the desorption experiments. In summary, 0.5 M HCl was used as a desorption agent to push out MB dye ions from the sorbent by adding the saturated PZB or PTB, or PZTB into 100 mL of 0.5 M HCl solution and shaking by an orbital shaker of 150 rpm for 2 h. After that, they were filtrated, rinsed with DI water, and air-dried, then they were ready for the next adsorption cycle. Equation (3) was used to calculate the desorption efficiency in the percentage.

3 $${\text{Desorption}}\,{\text{efficiency}}\,\left( \% \right)\, = \,\left( {q_{{\text{d}}} /q_{{\text{a}}} } \right)\, \times \,{1}00$$

where *q*_d_ is the amount of MB dye desorbed (mg/L) and *q*_a_ is the amount of MB dye sorbed (mg/L).

### Sorption isotherms and kinetics

The sorption isotherms and kinetics of PZB, PTB, and PZTB were studied by using several nonlinear isotherm models of Langmuir, Freundlich, Temkin, Dubinin-Radushkevich and nonlinear kinetic models of pseudo-first-order kinetic, pseudo-second-order kinetic, Elovich, intraparticle diffusion for determining which models were good to describe their sorption patterns or mechanisms. Equations (4), (5), (6), (7), (8), (9), (10), (11) demonstrated nonlinear equations of sorption isotherms and kinetics^2,21–29^.

4 $${\text{Langmuir:}}\,q_{e} = q_{{\text{m}}} K_{{\text{L}}} C_{{\text{e}}} /{1} + K_{{\text{L}}} C_{{\text{e}}}$$

where *q*_e_ is the capacity of dye sorption on sorbent at equilibrium (mg/g), *q*_m_ is the maximum amount of dye sorption on sorbent (mg/g),* C*_e_ is the equilibrium of dye concentration (mg/L), and* K*_L_ is Langmuir sorption constant (L/mg).

5 $${\text{Freundlich:}}\, q_{{\text{e}}} = K_{{\text{F}}} C_{{\text{e}}}^{{{1}/n}}$$

where *K*_F_ is the Freundlich constant of sorption capacity (mg/g)(L/mg)^1/n^ and *n* is the constant depicting the sorption intensity.

6 $${\text{Temkin:}}\,q_{{\text{e}}} = RT/b_{{\text{T}}} {\text{ln}}A_{{\text{T}}} C_{{\text{e}}}$$

where* R* is the universal gas constant (8.314 J/mol), *T* is the absolute temperature (K), *b*_T_ is the constant related to the heat of sorption (J/mol), and* A*_T_ is the equilibrium binding constant corresponding to maximum binding energy (L/mg).

7 $${\text{Dubinin - Radushkevich:}}\, q_{e} = q_{{\text{m}}} {\text{exp}}\left( { - K_{{{\text{DR}}}} \varepsilon^{{2}} } \right)$$

where* K*_DR_ is the activity coefficient related to mean sorption energy (mol^2^/J^2^), and *ε* is the Polanyi potential (J/mol).

8 $${\text{Pseudo - first - order:}}\,q_{t} \, = \,q_{e} \left( {1 - e^{{ - k_{1} t}} } \right)$$

where *q*_t_ is the amount of sorbed dye at the time (*t*) (mg/g). *k*_1_ is pseudo-first-order and pseudo-second-order rate constant (1/min).

9 $${\text{Pseudo - second - order:}}\, q_{{\text{t}}} = k_{{2}} q_{{\text{e}}}^{{2}} t/\left( {{1} + \, q_{{\text{e}}} k_{{2}} t} \right)$$

where* k*_2_ is pseudo-second-order rate constant (g/mg∙min).

10 $${\text{Elovich:}}\, q_{t} \, = \,\beta \,{\text{ln}}\,t\, + \,\beta \,{\text{ln}}\,\alpha$$

where* α* is the initial sorption rate (mg/g∙min) and *β* is the extent of surface coverage (g/mg).

11 $${\text{Intraparticle}}\,{\text{diffusion:}}\, q_{{\text{t}}} \, = \,k_{{\text{i}}} t^{{0.{5}}} \, + \,C_{{\text{i}}}$$

where *k*_i_ is the intraparticle diffusion rate constant (mg/g∙min^0.5^) and *C*_i_ is the constant that gives an idea about the thickness of the boundary layer (mg/g).

For sorption isotherms, 0.1 g of PZB or 0.1 g of PTB, or 0.08 g of PZTB was added into 250 mL of Erlenmeyer flask containing 100 mL of different MB dye concentrations from 25 to 200 mg/L, adjusted at pH 7, and tested the MB dye sorption by an incubator shaker at 25 °C for PZTB or 30 °C for PZB and PTB with a shaking speed of 150 rpm for 12 h.

For sorption kinetics, 1 g of PZB or 1 g of PTB, or 0.8 g of PZTB was added into 1000 mL of breaker containing 1000 mL of 100 mg/L of MB dye concentration, adjusted at pH 7, and tested the MB dye sorption by a jar test (JAR-TESTER, SF6, South Korea) with a shaking speed of 150 rpm for 15 h.

### Thermodynamic studies

The changing temperature might affect MB dye sorptions of PZB, PTB, and PZTB, so the thermodynamic studies were designed to examine how much it affects by using Eqs. (12), (13), (14) to calculate thermodynamic parameters^30^.

12 $$\Delta G^{ \circ } \, = \, - \,RT{\text{ln}}K_{{\text{c}}}$$

where *R* is the universal gas constant (8.314 J/mol K), *T* is the absolute temperature (K), and *K*_c_ is the equilibrium constant (L/mg).

13 $${\text{ln}}\,K_{{\text{c}}} \, = \, - \Delta H^{ \circ } /RT\, + \,\Delta S^{{^{ \circ } }} /{\text{R}}$$

14 $$\Delta G^{ \circ } \, = \,\Delta H^{ \circ } \, - \,T\Delta S^{ \circ }$$

where ∆*H*° and ∆*S*° values were calculated from the slope and intercept of the linear graph between ln *K*_c_ (*K*_c_ = *q*_e_/*C*_e_) and 1/*T*.

For the thermodynamic study, 0.1 g of PZB or 0.1 g of PTB, or 0.08 g of PZTB were added to 250 mL of Erlenmeyer flask containing 100 mL of 100 mg/L MB dye concentration, adjusted at pH 7, and tested the MB dye sorption by an incubator shaker with different temperatures from 293.15 to 323.15 K with a shaking speed of 150 rpm at 12 h.

## Result and discussion

### The physical characteristics

**Figure 2:**
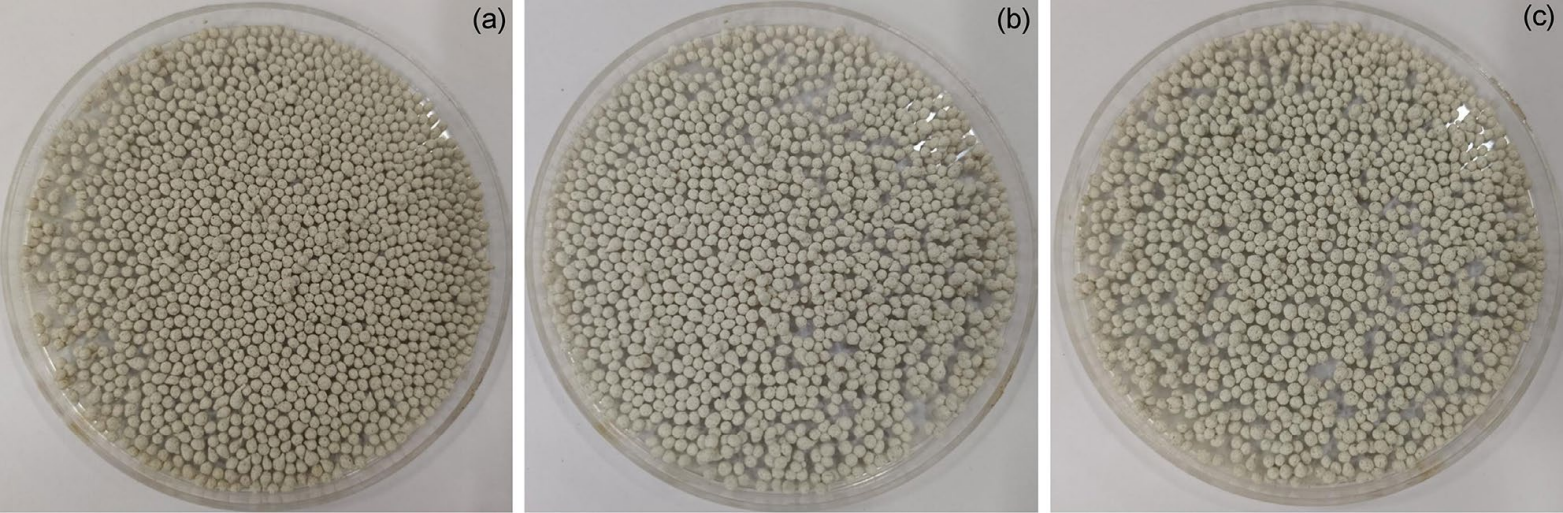
The physical characteristics of (**a**) PZB, (**b**) PTB, and (**c**) PZTB.

The physical characteristic images of PZB, PTB, and PZTB were demonstrated in Fig. 2a–c which had a spherical shape. They had different colors depending on the type of metal oxide added to pomelo beaded sorbents. PZB had cream-colored beads, whereas PTB had white-colored beads. For PZTB, it had light cream color beads which was a color between PZB and PTB.

### Sorbent characterizations

#### X-Ray Diffractometer (XRD)

**Figure 3:**
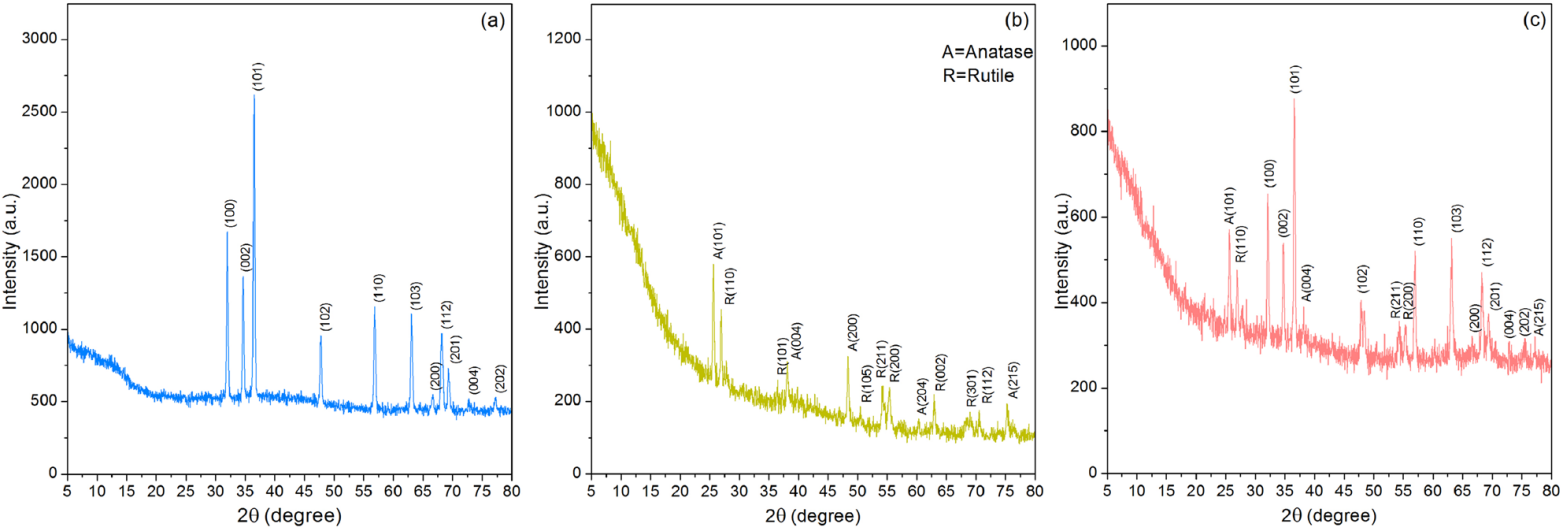
The crystalline structures of (**a**) PZB, (**b**) PTB, and (**c**) PZTB.

Figure 3a–c demonstrated the results of XRD analysis to determine the crystalline structures of PZB, PTB, and PZTB which had amorphous structures with specific peaks of each metal oxide in sorbents. The specific peaks of zinc oxide were found in PZB and PZTB at 2ϴ of 32.03° (100), 34.68° (002), 36.50° (101), 47.81° (102), 56.83° (110), 63.07° (103), 66.45° (200), 68.19° (112), 68.30° (201), 72.53° (004), and 76.46° (202) corresponded to JCPDS no. 36–1451 similar found in previous studies^19,21,31^ shown in Fig. 3a and c. The specific peaks of titanium dioxide were found in PTB consisted of two phases which were the anatase phase at 2ϴ of 25.63° (101), 38.14° (004), 48.35° (200), 60.16° (204), and 75.29° (215) matched to JCPDS no. 21–1272^32^ and the rutile phase at 2ϴ of 26.96° (110), 36.47° (101), 50.49° (105), 54.13° (211), 55.37° (220), 62.97° (002), 68.99° (301), and 70.56° (112) related to JCPDS no.21–1276^32^ displayed in Fig. 3b. While PZTB found the speck peaks of titanium dioxide in the anatase phase at 2ϴ of 25.63° (101), 38.14° (004), and 75.29° (215) and the rutile phase at 2ϴ of 26.96° (110), 54.13° (211), and 55.37° (220) displayed in Fig. 3c. As a result, metal oxides of zinc and titanium could be added to pomelo beaded sorbents which they are bound and retained in the structures of PZB, PTB, and PZTB bringing about specific functionality of the composites.

#### Field Emission Scanning Electron Microscopy and Focus Ion Beam (FESEM-FIB)

**Figure 4:**
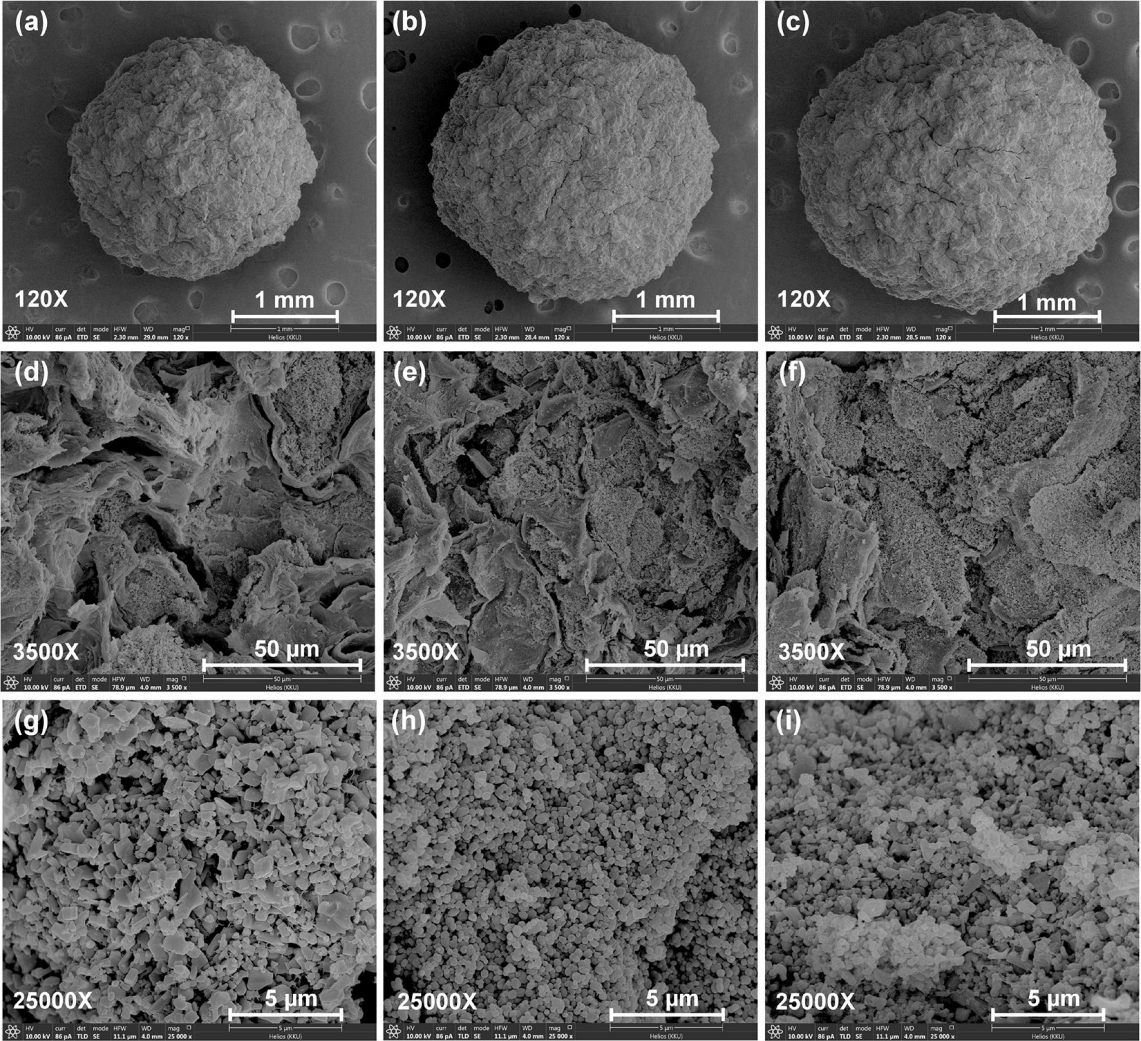
FESEM-FIB images of (**a**,**d**,**g**) PZB, (**b**,**e**,**h**) PTB, and (**c**,**f**,**i**) PZTB.

The FESEM-FIB images revealed the surface morphologies of PZB, PTB, and PZTB at 120X magnification with 1 mm, 3500X magnification with 50 µm, and 25,000X magnification with 5 µm for the spherical shape, surface by a cross-section, and metal oxide zooming by a cross-section, respectively shown in Fig. 4a–i. For a spherical shape, they were heterogeneous surfaces shown in Fig. 4a–c. For the surface, they had lamella structures with coarse surfaces that corresponded to the pomelo peel structure found in a previous study^11^ demonstrated in Fig. 4d–f. For metal oxide zooming, zinc oxide (ZnO) was found in PZB which had a square shape similar found in a previous study^33^ shown in Fig. 4g. While titanium dioxide (TiO_2_) was found in PTB which had a circular shape similar reported by a previous study^34^ illustrated Fig. 4h. For PZTB, the distribution of ZnO and TiO_2_ was found on its surface demonstrated in Fig. 4i.

#### Energy Dispersive X-Ray Spectrometer (EDX)

**Figure 5:**
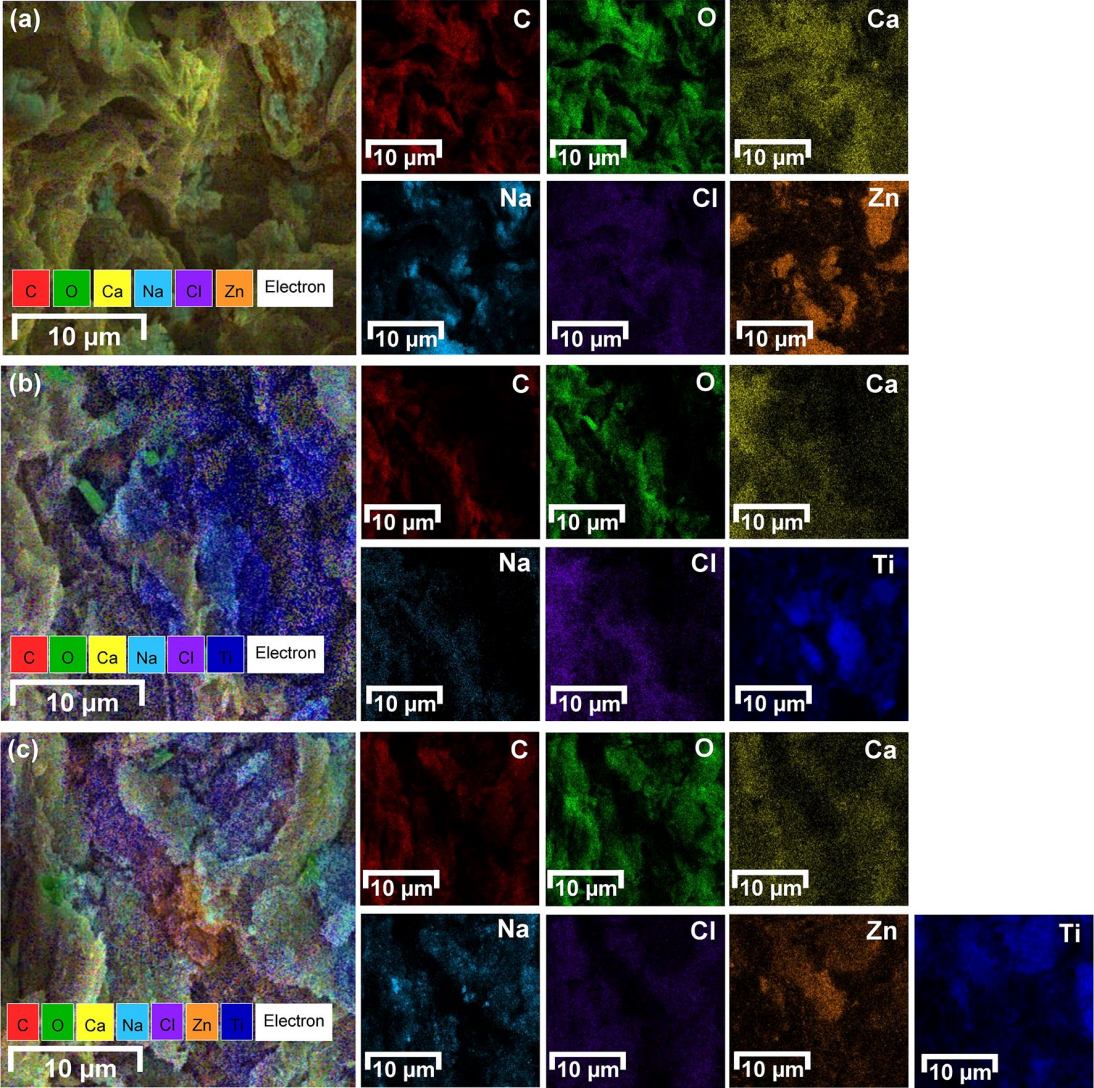
The chemical mapping distributions of (**a**) PZB, (**b**) PTB, and (**c**) PZTB.

PZB, PTB, and PZTB had five main chemical elements of carbon (C), oxygen (O), calcium (Ca), sodium (Na), and chloride (Cl), whereas zinc (Zn) or titanium (Ti) also detected them with close percentage by weight of 1:1 following a ratio metal oxide added in the synthesis method shown in Table 2. Moreover, their elemental mapping distributions were illustrated in Fig. 5a–c with the main elements mentioned above distributed on their surfaces. In addition, Zn and Ti were also distributed throughout their surfaces confirming the addition of two metal oxides into pomelo beaded sorbents.

**Table 2:** The chemical compositions of PZB, PTB, and PZTB.

Sorbents	C	O	Ca	Na	Cl	Zn	Ti
PZB	35.3	27.4	4.6	0.9	2.3	29.5	–
PTB	35.7	28.6	4.7	0.7	2.6	–	27.7
PZTB	26.6	23.5	2.8	0.8	1.8	23.9	20.6

#### Fourier Transform Infrared Spectroscopy (FT-IR)

**Figure 6:**
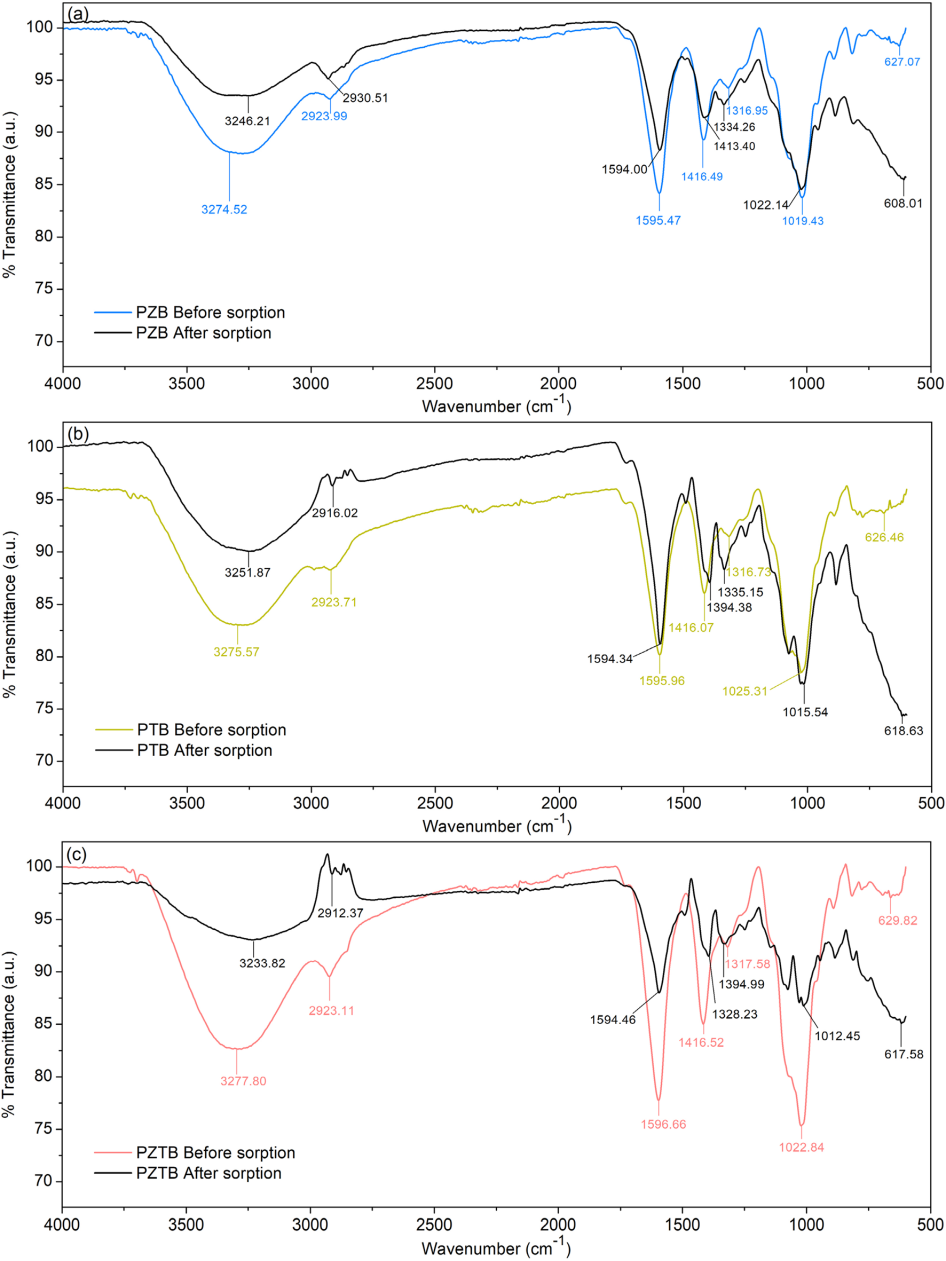
FT-IR spectra of (**a**) PZB, (**b**) PTB, and (**c**) PZTB.

Figure 6a–c displayed the chemical functional groups of PZB, PTB, and PZTB which had four main functional groups consisting of hydroxyl (O–H), methyl and methylene (C–H), carbonyl (C=O) or (C–O–C), and carboxyl (C–O). Furthermore, the specific peaks of zinc oxide (ZnO) or titanium dioxide (TiO_2_) were also demonstrated in pomelo beaded sorbents. In the wavenumbers of 3200 and 1300 cm^−1^, they represented O–H of pectin, cellulose, lignin, carboxylic acids, and phenol compounds^11^. For the wavenumber of 2900 cm^−1^, it displayed C–H in methyl (CH_3_) and methylene (CH_2_) groups^35^. The wavenumber of 1500 cm^−1^ referred to C=O of carboxylic acids in lignin and sodium alginate^36^. C–O presented ionic carboxylic groups found in the wavenumber of 1500 cm^−1^^37^. For C–O–C, it showed the sodium alginate and anhydroglucose of cellulose or hemicellulose found in the wavenumber of 1000 cm^−1^^36^. Moreover, the specific peaks of zinc oxide found in PZB and PZTB displayed in the form of Zn–O in wavenumber of 627 and 629 cm^−1^, and the specific peaks of titanium dioxide found in PTB and PZTB shown in the form of Ti–O–Ti in wavenumber of 626 and 629 cm^−1^^2^. The pomelo peels and sodium alginate were formed through an ion exchange process^38^, and then their hydroxyl groups (O–H) interacted with the hydroxyl groups (O–H) in ZnO or TiO_2_ through hydrogen bonds to become composited sorbents (Zn–O/Ti–O–Ti–C=O–OH)^39^. After MB dye sorptions, the intensity peaks in their chemical functional groups especially O–H and C=O positions were decreased with a little bit of shift of wavelengths. In addition, the low-frequency bands of Zn–O and Ti–O–Ti were detected. These might be evidence of their MB dye sorptions similar reported in the previous study^40^.

#### The point of zero charge (pH_pzc_)

**Figure 7:**
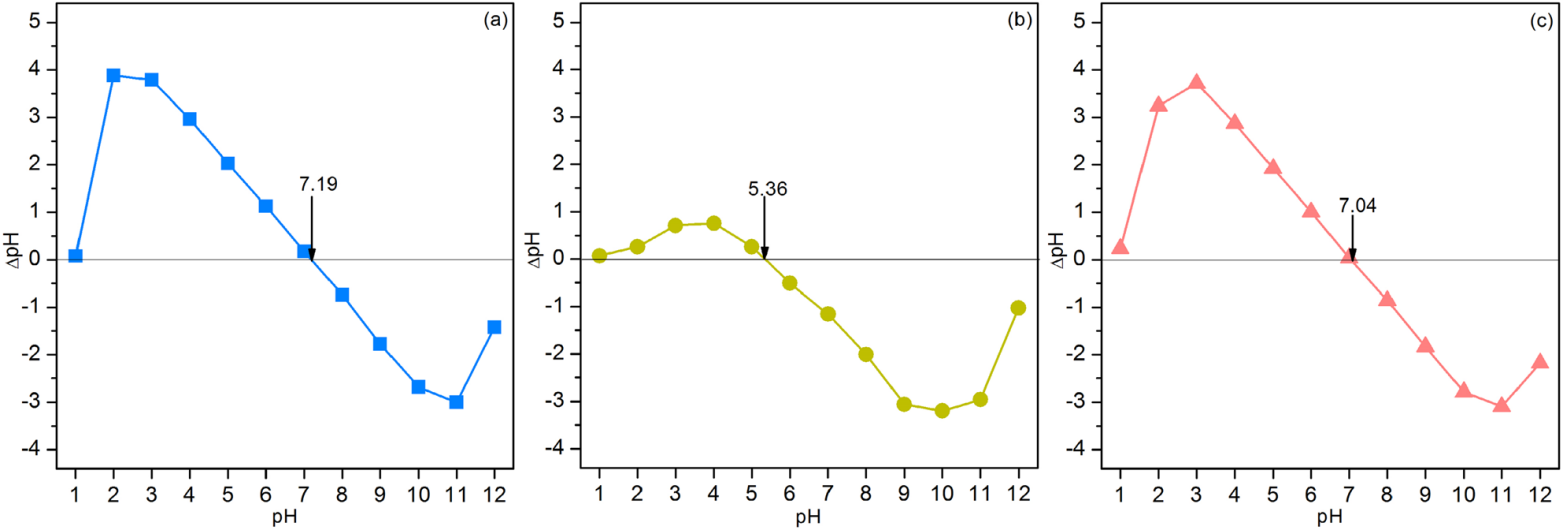
The points of zero charge of (**a**) PZB, (**b**) PTB, and (**c**) PZTB.

Figure 7a–c illustrated the results of the points of zero charge of PZB, PTB, and PZTB which were 7.19, 5.36, and 7.04, respectively. The addition of zinc oxide into pomelo beaded sorbents had a higher pH_pzc_ than titanium dioxide, whereas the addition of both metal oxides had a middle pH_pzc_ between each other. As a result, their MB dye sorptions should occur at pH solution more than their pH_pzc_.

### Batch experiments

**Figure 8:**
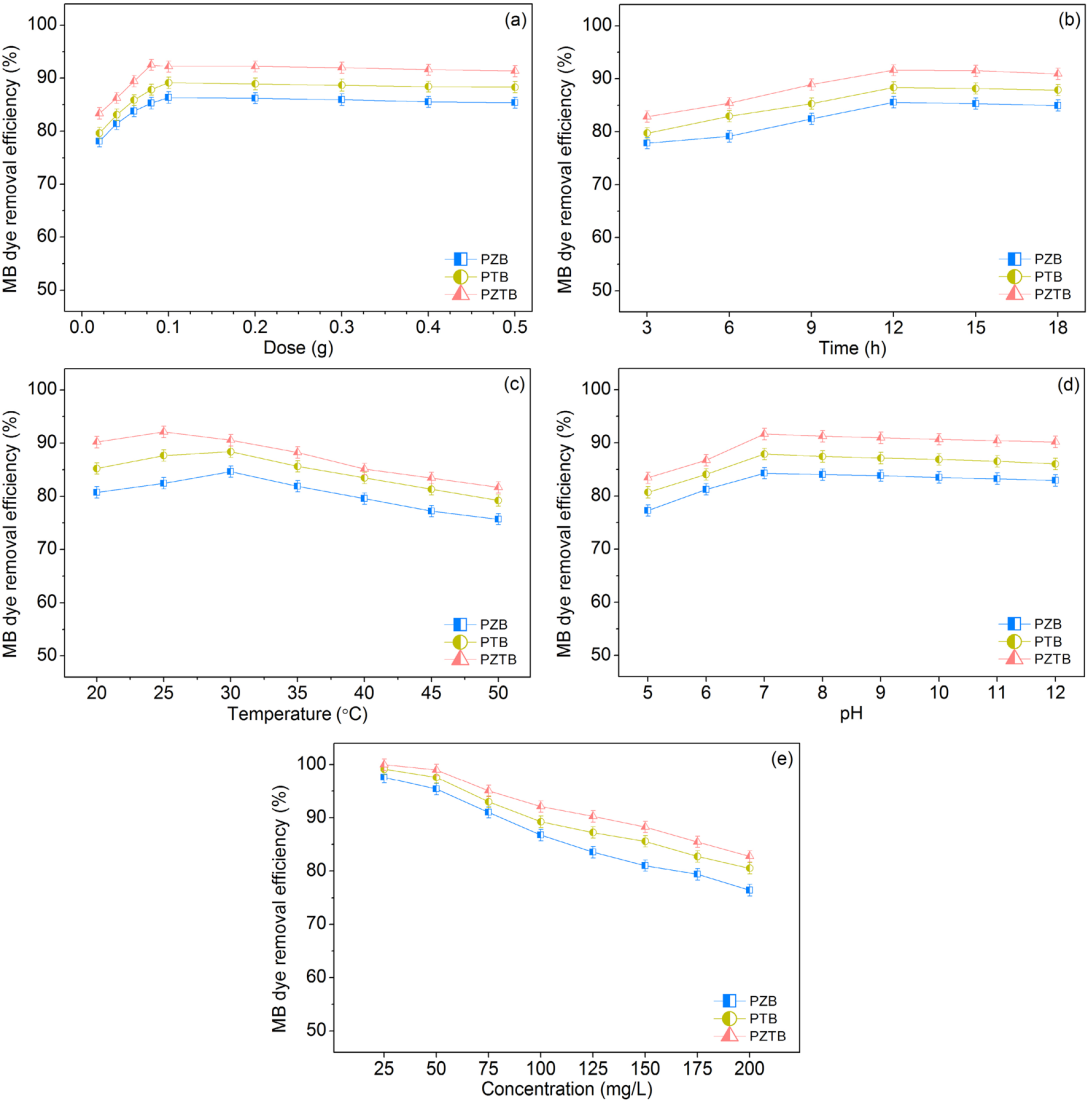
MB dye sorptions by batch experiments on the effects of (**a**) dose, (**b**) time, (**c**) temperature, (**d**) pH, and (**e**) concentration of PZB, PTB, and PZTB.

The sorbent efficiencies for MB dye sorptions of PZB, PTB, and PZTB were investigated by batch experiments, and the results were illustrated in Fig. 8a–e. For the effect of dosage, the increasing dosage resulted in the increases of their MB dye removal efficiencies from 0.02 to 0.1 g, and then they were constant. This might be from the increasing dosage is the increase of active site, so the sorbent can be more sorbed dye ions^41^. However, the highest dye removal efficiency does not exceed the equilibrium sorption of sorbent which observes the dye removal efficiency to be constant^19,31^. In addition, the highest MB dye removal efficiencies of PZB, PTB, and PZTB were found at 0.1 g (86.33%), 0.1 g (89.15%), and 0.08 g (92.52%), respectively. For the effect of contact time, the increasing contact times affected their increasing MB dye removal efficiencies from 3 to 12 h because the sorbents had a long time to sorb MB dye. After 12 h, their MB dye removal efficiencies were constant because they had gone into the equilibrium sorptions related to the saturation of their active sites^42^. Thus, the contact time of 12 h represented the equilibrium contact time of all sorbents because it demonstrated their highest MB dye removal efficiencies of PZB, PTB, and PZTB at 12 h for 85.55%, 88.33%, and 91.60%, respectively. For the effect of temperature, the increasing temperature resulted in the increase of MB dye removal efficiencies from 20 to 30 °C, and then they decreased. As a result, they did not favor MB dye sorptions at high temperatures caused by the changing of sorbent structures such as cellulose, and lignin^43^. Moreover, the highest MB dye removal efficiencies of PZB, PTB, and PZTB were found at 30 °C (84.63%), 30 °C (88.37%), and 25 °C (92.07%), respectively. For the effect of pH, the increasing pH resulted in the increase of MB dye removal efficiencies from pH 5–7, and then they were constant. At pH 5–6, the surfaces of PZB, PTB, and PZTB were positively charged, so they had low MB dye removal efficiencies because of the force of repulsion between charges of the same type. At pH 7–12, their surfaces were negatively charged, so they had high MB dye removal efficiencies. Furthermore, the highest MB dye removal efficiencies of PZB, PTB, and PZTB were found at pH 7 for 84.29%, 87.88%, and 91.63%, respectively which corresponded with their MB dye sorptions should occur at pH_solution_ > pH_pzc_. For the effect of concentration, the increasing concentration resulted in decreasing MB dye removal efficiencies because of the decrease of the active site of sorbents. Therefore, the optimum conditions of dosage, contact time, temperature, pH, and concentration of PZB, PTB, and PZTB were 0.1 g, 12 h, 30 °C, pH 7, and 100 mg/L for 86.75%, 0.1 g, 12 h, 30 °C, pH 7, and 100 mg/L for 89.26%, and 0.08 g, 12 h, 25 °C, pH 7, and 100 mg/L for 92.11%, respectively. Moreover, they could be arranged from high to low MB dye removal efficiency to be PZTB > PTB > PZB, and PZTB illustrated the highest MB dye removal efficiency than other sorbents with not only offering high MB dye removal efficiency but also spending less dosage and temperature than others. Therefore, the pomelo beaded sorbents with ZnO or TiO_2_ modifications increased sorbent efficiency especially the mixture of ZnO and TiO_2_ in a ratio of 1:1.

### Sorbents reusability studies

**Figure 9:**
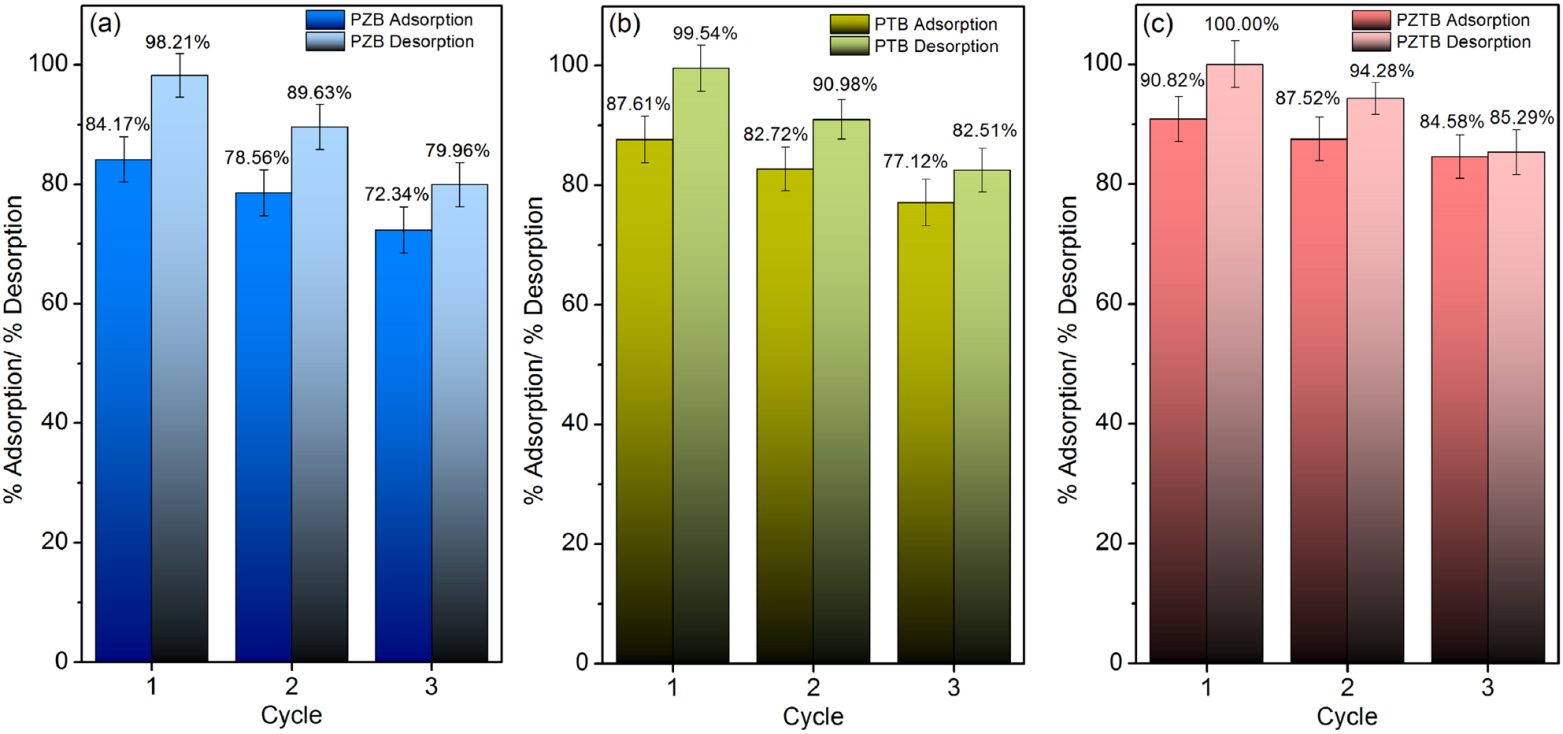
The desorption experiments of (**a**) PZB, (**b**) PTB, and (**c**) PZTB.

The sorbent reusability was studied to investigate how many cycles PZB, PTB, and PZTB were potential MB dye sorptions through the desorption experiments using three sorption–desorption cycles to estimate the cost-effectiveness of sorbents before use in industrial wastewater treatment. The results were illustrated in Fig. 9a–c, and they could be adsorbed with MB dye for more than three cycles with more than 72%. For PZB, the sorption–desorption in three cycles were in ranges of 72.34–84.17% and 79.96–98.21% which were decreased by approximately 12% and 18%. For PTB, the sorption–desorption in three cycles were in ranges of 77.12–87.61% and 82.51–99.54% which were decreased by approximately 10% and 17%. For PZTB, the sorption–desorption in three cycles were in ranges of 84.58–90.82% and 85.29–100% which were decreased by approximately 6% and 15%. Therefore, pomelo beaded sorbents were good for application in industrial wastewater treatment offering cost-effective investment.

### Sorption isotherms

**Figure 10:**
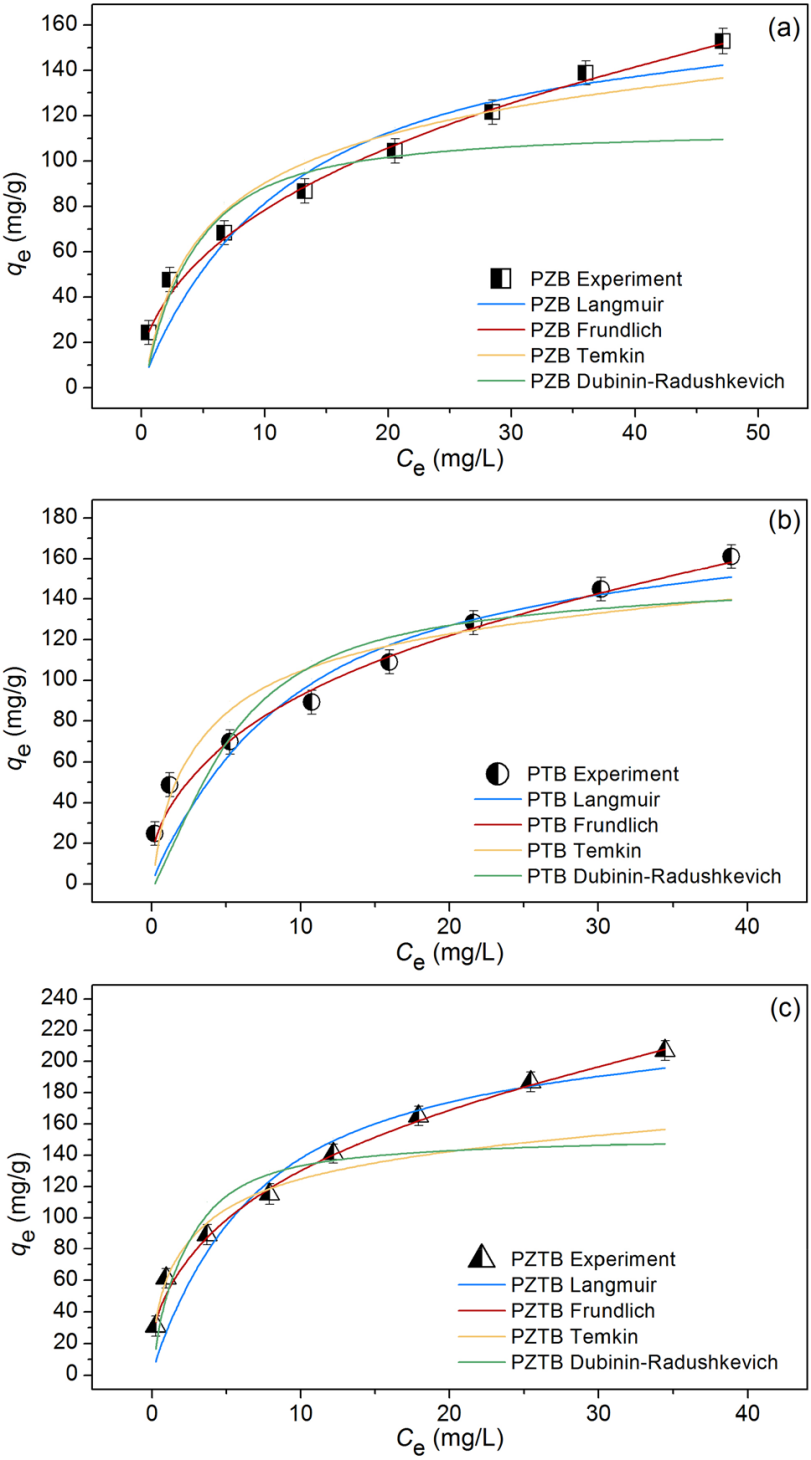
Graphs of Langmuir, Freundlich, Temkin, and Dubinin-Radushkevich isotherms of (**a**) PZB, (**b**) PTB, and (**c**) PZTB for MB dye sorptions.

The results of sorption isotherms of PZB, PTB, and PZTB were displayed in Fig. 10a–c, and Table 3 illustrated their isotherm parameters. Since the highest *R*^2^ value is a good fit model to describe the sorption pattern, the Freundlich model was chosen because it presented the highest *R*^2^ close to 1 in all sorbents compared to other isotherm models. As a result, their sorption patterns are related to physiochemical sorption which might be from the addition of zinc oxide or titanium dioxide into pomelo beaded sorbents similar reported by other studies^2,21,44^. For 1/*n* and *K*_F_ comparison, since their 1/*n* values were in a range of 0 < 1/*n* < 1, they favorably sorbed MB dye. Moreover, PZTB had a higher Freundlich constant of sorption capacity (*K*_F_) than other sorbents, so it was possible more MB dye sorption than PZB and PTB.

**Table 3:** The isotherm parameters for MB dye sorptions on PZB, PTB, and PZTB**.**

Isotherm model	Parameter	PZB	PTB	PZTB
Langmuir	*q*_m_ (mg/g)	174.830	186.201	233.713
	*K*_L_ (L/mg)	0.093	0.110	0.150
	*R*^2^	0.937	0.905	0.927
	*R*^2^_adj_	0.927	0.890	0.914
	RMSE	12.112	15.807	18.061
Freundlich	1*/n*	0.415	0.386	0.371
	*K*_F_ (mg/g)(L/mg)^1/n^	30.724	38.542	55.759
	*R*^2^	0.996	0.990	0.997
	*R*^2^_adj_	0.996	0.989	0.996
	RMSE	2.923	5.073	3.757
Temkin	*b*_T_ (J/mol)	88.203	100.755	101.366
	*A*_T_ (L/g)	2.542	6.900	15.698
	*R*^2^	0.932	0.895	0.815
	*R*^2^_adj_	0.921	0.878	0.785
	RMSE	12.611	16.625	28.662
Dubinin-Radushkevich	*q*_m_ (mg/g)	115.435	153.493	153.238
	*K*_DR_ (mol^2^/kJ^2^)	0.001	0.001	0.001
	*E* (kJ/mol)	22.506	18.291	30.209
	*R*^2^	0.948	0.815	0.946
	*R*^2^_adj_	0.939	0.784	0.937
	RMSE	16.830	22.134	33.474

The comparison of the maximum sorption capacity (*q*_m_) of pomelo sorbents with or without modifications of thermal, chemical, and metal for MB dye sorptions is demonstrated in Table 4 which PZTB had a higher *q*_m_ than others. While PZB and PTB had higher *q*_m_ than pomelo peel biochar at 500 °C and 600 °C and pomelo peel modified by iron(III) chloride. Therefore, they were potential beaded sorbents for removing MB dye in an aqueous solution, especially PZTB.

**Table 4:** Comparison of the maximum sorption capacity (*q*_m_) of pomelo sorbents for MB dye sorption.

Sorbents	Temperature (°C)	pH	Concentration (mg/L)	*q*_m_ (mg/g)	References
Pomelo fruit peel	30	10	5–1000	218.50	^11^
Pomelo peel biochar (500 °C)	25	7	20–200	28.65	^13^
Pomelo peel biochar (600 °C)	25	7	0.5–10	154.71	^14^
Pomelo peel biochar (600 °C) modified by nitric acid	25	7	0.5–10	176.20	^14^
Pomelo peel biochar (600 °C) modified by sulfuric acid	25	7	0.5–10	209.16	^14^
Pomelo peel modified by iron(III) chloride	25	12	20–300	79.47	^15^
PZB	30	7	25–200	174.83	This study
PTB	30	7	25–200	186.20	This study
PZTB	25	7	25–200	233.71	This study

### Sorption kinetics

**Figure 11:**
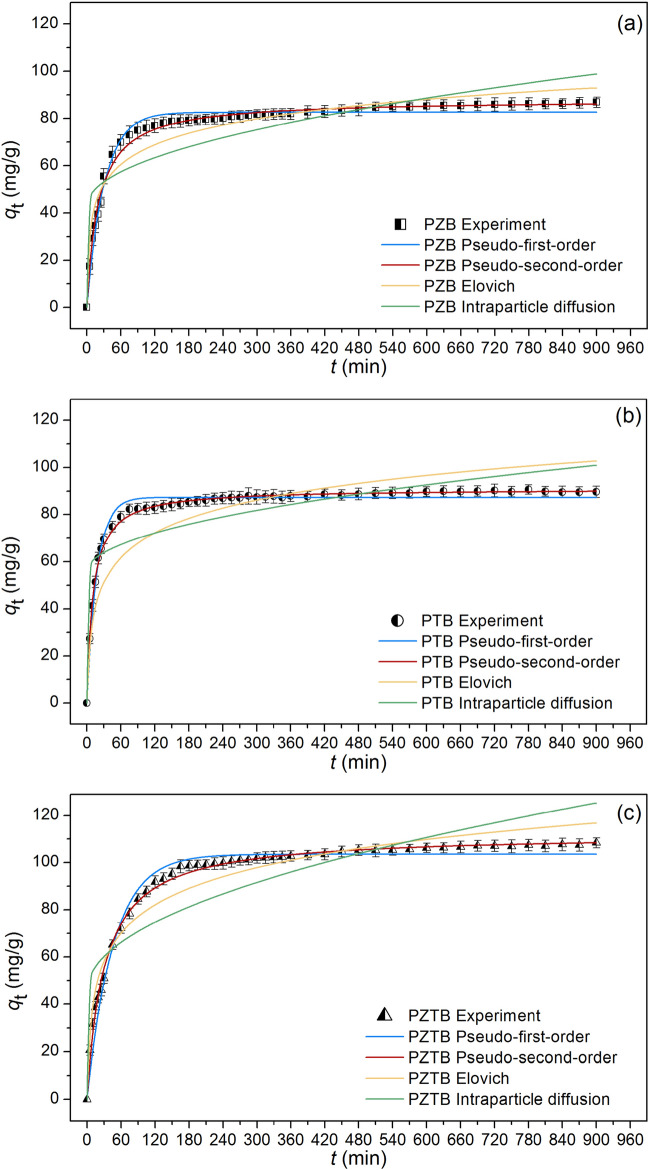
Graphs of pseudo-first-order kinetic, pseudo-second-order kinetic, Elovich, and intraparticle diffusion models of (**a**) PZB, (**b**) PTB, and (**c**) PZTB for MB dye sorptions.

The results of the sorption kinetics of PZB, PTB, and PZTB were demonstrated in Fig. 11a–c and Table 5 displayed their kinetic parameters. Since the *R*^2^ values of the pseudo-second-kinetic model were higher than the *R*^2^ values of other kinetic models with close to 1, their rates and mechanisms were well described by the pseudo-second-kinetic model relating to a chemical sorption process similar to other studies reported^2,21,44^. Moreover, RMSE is also another value used for deciding the good-fit kinetic model, and the low RMSE is chosen. In Table 6, since the RMSE in the pseudo-second-kinetic model demonstrated the lowest value in all sorbents than other models, their rates and mechanisms corresponded with the pseudo-second-kinetic model agreed with the results of *R*^2^. For *q*_e_ and *k*_2_ comparisons, PZTB had a higher *q*_e_ than other sorbents, so it could have more ability to sorb MB dye than others. While the *k*_2_ value of PTB was higher than other sorbents, it had a higher sorption MB dye rate than others.

**Table 5:** The kinetic parameters of PZB, PTB, and PZTB for MB dye sorptions.

Kinetic model	Parameter	PZB	PTB	PZTB
Pseudo-first-order	*q*_e_ (mg/g)	82.582	87.298	103.521
	*k*_1_ (1/min)	0.033	0.058	0.022
	*R*^2^	0.979	0.978	0.977
	*R*^2^_adj_	0.978	0.978	0.976
	RMSE	2.888	2.647	4.083
Pseudo-second-order	*q*_e_ (mg/g)	87.958	90.949	112.059
	*k*_2_ (g/mg·min)	0.0006	0.0010	0.0003
	*R*^2^	0.992	0.997	0.995
	*R*^2^_adj_	0.992	0.997	0.995
	RMSE	1.724	1.029	1.822
Elovich	*α* (mg/g·min)	2.673	1.000	0.991
	*β* (g/mg)	11.934	15.105	17.191
	*R*^2^	0.917	0.716	0.944
	*R*^2^_adj_	0.915	0.710	0.942
	RMSE	6.136	10.268	6.813
Intraparticle diffusion	*k*_i_ (mg/g·min^0.5^)	1.863	1.510	2.655
	*C*_i_ (mg/g)	42.950	55.650	45.633
	*R*^2^	0.627	0.517	0.701
	*R*^2^_adj_	0.618	0.507	0.695
	RMSE	13.018	13.389	15.692

**Table 6:** Thermodynamic parameters of PZB, PTB, and PZTB for MB dye sorptions.

Sorbents	Δ*G*˚ (kJ/mol)							Δ*H*˚ (kJ/mol)	Δ*S*˚ (J/mol K)
	293.15 (K)	298.15 (K)	303.15 (K)	308.15 (K)	313.15 (K)	318.15 (K)	323.15 (K)		
PZB	− 5.34	− 4.74	− 4.52	− 3.81	− 3.43	− 2.95	− 2.71	− 31.63	− 89.90
PTB	− 6.03	− 5.63	− 5.11	− 4.57	− 4.22	− 3.89	− 3.60	− 30.55	− 83.81
PZTB	− 7.14	− 6.63	− 6.25	− 5.73	− 5.12	− 4.87	− 4.61	− 32.79	− 87.69

### Thermodynamic studies

**Figure 12:**
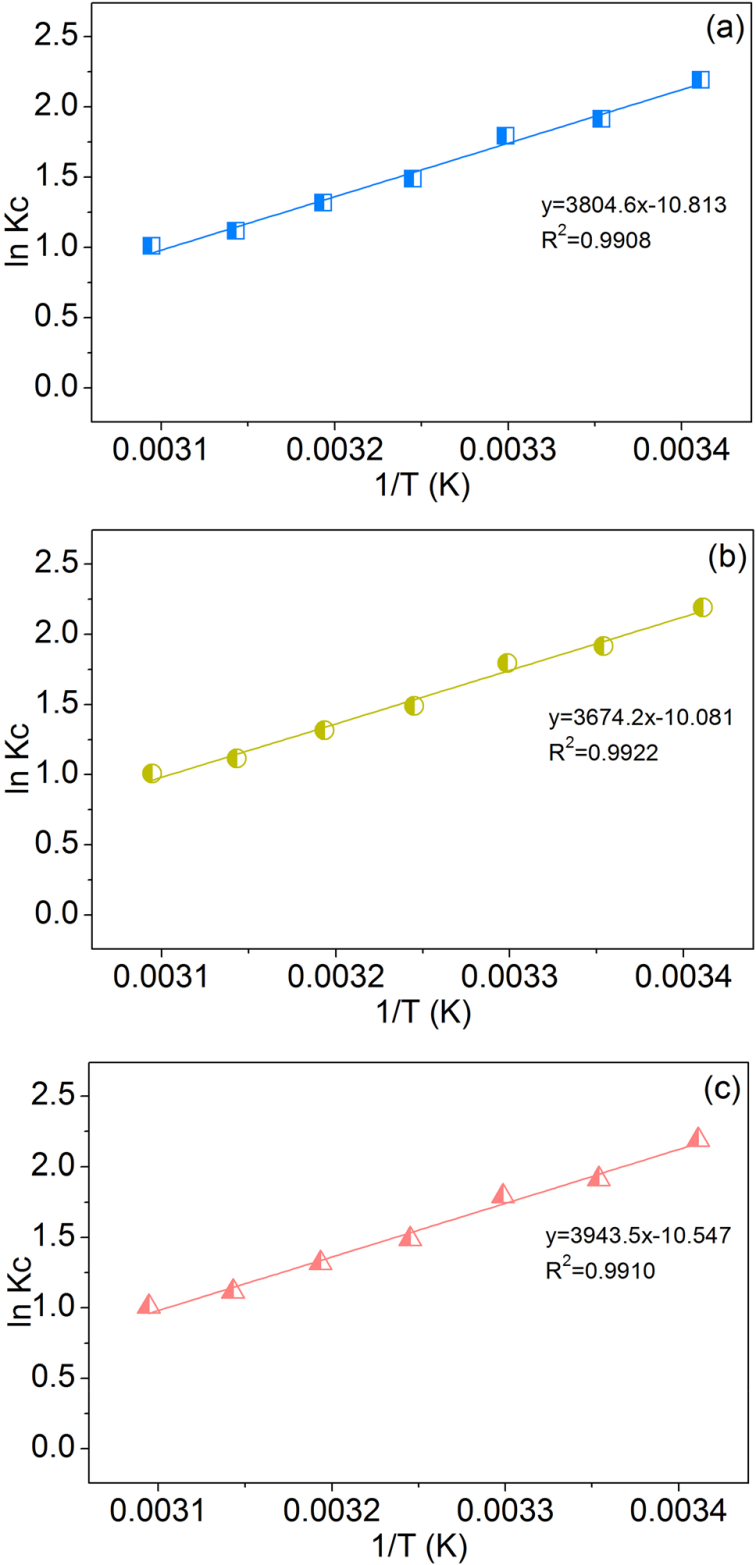
Thermodynamic plots of (**a**) PZB, (**b**) PTB, and (**c**) PZTB for MB dye sorptions.

The thermodynamic study is used for investigating how much temperature affects MB dye sorption by sorbent and to know whether its sorption process can naturally occur. Table 6 and Fig. 12a–c demonstrated their thermodynamic results. Since their ∆*G*° values were negative, their MB dye sorptions could naturally occur. In addition, their sorption processes were exothermic processes they did not favor MB dye sorptions with increasing temperature because of their negative ∆*H*° values. Moreover, the ∆*H*° value can also indicate the sorption process that the sorbent is holding. If ∆*H*° is in the range of (− 20)–(0) kJ/mol, it is a process of physic sorption. If ∆*H*° is in the range of (− 80)–(− 400) kJ/mol, it is a process of chemical sorption^45,46^. Since the ∆*H*° values of PZB, PTB, and PZTB were in the range (− 20)–(− 80) kJ/mol, they were both physical and chemical sorption processes. For ∆*S*˚, they were also negative which meant their sorption processes randomly decreased in solid solution interfaces.

## The feasible mechanisms of MB dye sorptions

**Figure 13:**
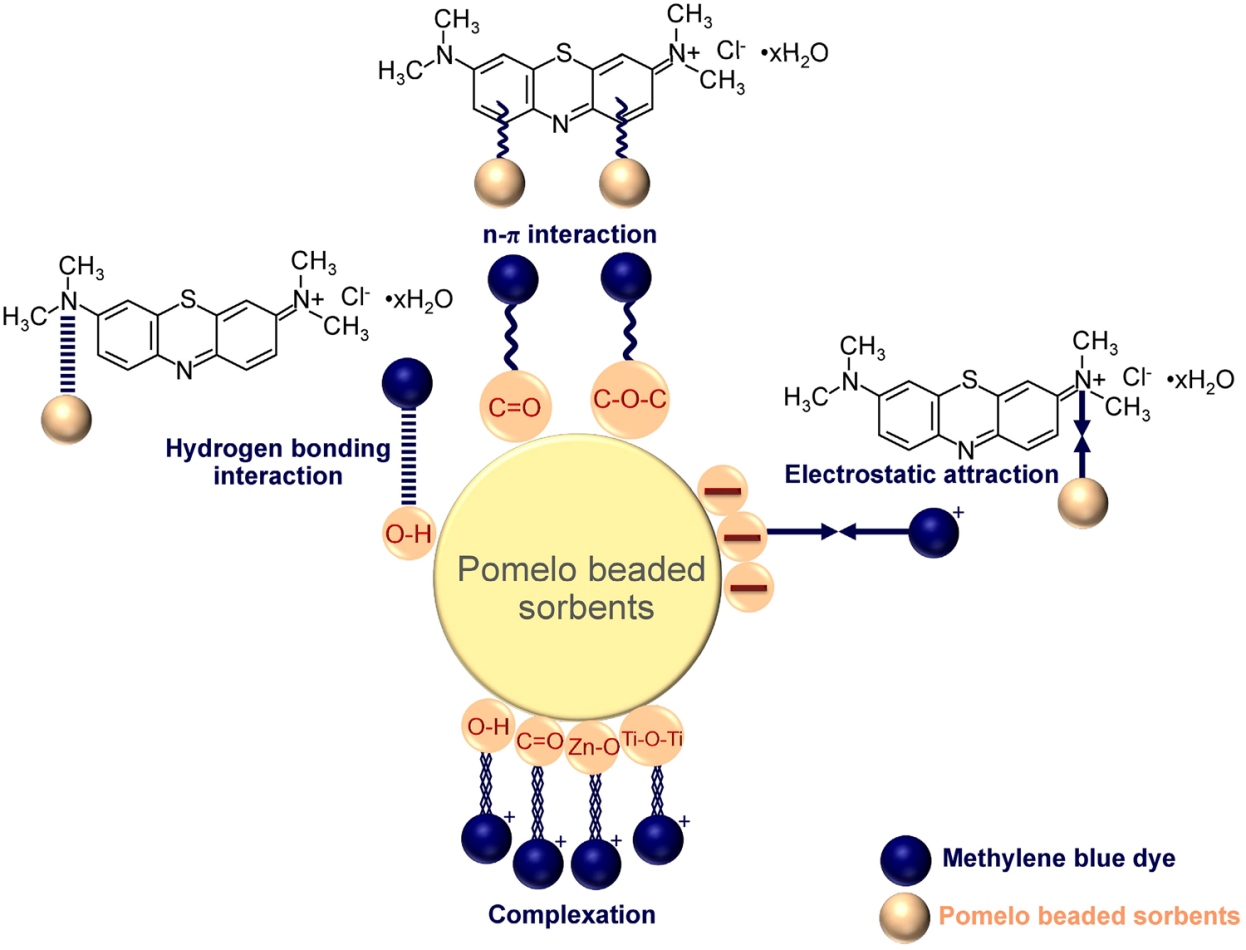
Feasible mechanisms of MB dye sorptions on PZB, PTB, and PZTB.

Both physisorption and chemisorption played important roles in describing feasible multi-mechanisms of MB dye sorptions by PZB, PTB, and PTZB modified an idea from Praipipat et al.^44^. Multi-mechanisms included the electrostatic attraction, hydrogen bonding interaction, n-π interaction, and complexation were demonstrated in Fig. 13. For electrostatic attraction, the negative charges of PZB, PTB, and PTZB at pH_solution_ > their pH_pzc_ interacted with MB dye ions^11^. For hydrogen bonding interaction, the hydroxyl groups (O–H) on their surfaces caught the nitrogen atom in the MB dye structure^47^. For n-π interaction, the carbonyl (C=O) or (C–O–C) on their surfaces interacted with the aromatic rings of MB dye^11^. For the complexation, O–H, C=O, Zn–O, and Ti–O–Ti functional groups in composited sorbents of PZB, PTB, and PZTB could catch the positively charged MB dye molecules through chemical affinity^48^.

## Conclusion

This study presented the performances of three pomelo beaded sorbents which were pomelo-doped zinc oxide beads (PZB), pomelo-doped titanium dioxide beads (PTB), and pomelo-doped zinc oxide and titanium dioxide beads (PZTB) to eliminate MB dye in an aqueous solution. The successful additions of zinc oxide (ZnO) or titanium dioxide (TiO_2_) into PZB, PTB, and PZTB were confirmed by XRD, EDX, and FT-IR techniques which presented the specific peaks of ZnO or TiO_2_, chemical elements of Zn or Ti, and chemical functional groups of Zn–O or Ti–O–Ti. Moreover, their MB dye removal efficiencies could be arranged from high to low of PZTB > PTB > PZB. Since PZTB demonstrated the highest MB dye removal efficiency of 92.11%, the additions of both ZnO and TiO_2_ were recommended to increase the capacity of pomelo beaded sorbents. Furthermore, their sorption patterns and mechanisms corresponded to Freundlich and pseudo-second-order kinetic models, and they could be reused for more than three cycles. Their MB dye sorption mechanisms could be explained by multi-interactions of electrostatic attraction, hydrogen bonding interaction, n-π interaction, and complexation. Therefore, they are potential sorbents for sorbing MB dye in wastewater, especially PZTB.

Before industrial applications, the column experiments with continuing flow rate should be investigated, and the competing ions are interesting investigations to confirm the specific target sorption of PZB, PTB, and PZTB.

## Data Availability

The datasets used and/or analyzed during the current study are available from the corresponding author upon reasonable request.
